# BASELINE PURPOSE IN LIFE PREDICTS FUNCTIONAL HEALTH ACROSS 16 WEEKS IN OLDER ADULTS WITH PROGRESSIVE VISION LOSS

**DOI:** 10.1093/geroni/igac059.2902

**Published:** 2022-12-20

**Authors:** Wingyun Mak, Silvia Sörensen

**Affiliations:** The New Jewish Home Research Institute on Aging, New York, New York, United States; University of Rochester, Rochester, New York, United States

## Abstract

Greater purpose in life is associated with various positive outcomes in later life, such as, lower mortality risk, protection against cognitive decline, and lower spending on medical care and emergency room visits. Recent research from the Health and Retirement Study has shown that older adults with greater purpose in life have fewer physical limitations after four years. There has been no research to investigate purpose in life in older adults with progressive vision loss. The aim of this study is to examine whether purpose in life predicts functional health (i.e., activities of daily living) across a 16-week period of time in a sample of older adults (aged 60–96) with vision loss due to macular degeneration who were enrolled in a resilience building program (Nf 122). A repeated measures GLM showed that functional health did not change across 16 weeks (ns). Age (F(1, 112)=8.91, p=.003, ηp2=.07) and purpose in life (F(1,112)=13.21, p < .001, ηp2= .11) were significant overall predictors of functional health across time. Gender, education, cognition, marital status, depression, and loneliness at baseline were not associated with overall functional health. People who were younger were more likely to have better functional health over 16 weeks; purpose in life added 11% of explained variance. Because purpose in life is malleable and also significantly associated with better functional health in older adults with progressive vision loss, future interventions that enhance purpose in life should be studied with this population.

